# Genome-Wide Identification and Expression Analysis of the *BTB* Gene Superfamily Provides Insight into Sex Determination and Early Gonadal Development of *Alligator sinensis*

**DOI:** 10.3390/ijms251910771

**Published:** 2024-10-07

**Authors:** Pengfei Li, Peng Liu, Dongsheng Zang, Changcheng Li, Chong Wang, Yunzhen Zhu, Mengqin Liu, Lilei Lu, Xiaobing Wu, Haitao Nie

**Affiliations:** The Anhui Provincial Key Laboratory of Biodiversity Conservation and Ecological Security in the Yangtze River Basin, College of Life Science, Anhui Normal University, Wuhu 241000, China; 2221011668@ahnu.edu.cn (P.L.); liupeng@ahnu.edu.cn (P.L.); 1072888405@ahnu.edu.cn (D.Z.); lichangcheng@ahnu.edu.cn (C.L.); wangc@ahnu.edu.cn (C.W.); zyz2059368613@163.com (Y.Z.); 17856872220@163.com (M.L.); 18255539754@163.com (L.L.)

**Keywords:** *Alligator sinensis*, *BTB* gene superfamily, genome-wide identification, expression analyses, sex differentiation, gonadal development, evolutionary analyses

## Abstract

The *BTB* gene superfamily is widely distributed among higher eukaryotes and plays a significant role in numerous biological processes. However, there is limited knowledge about the structure and function of *BTB* genes in the critically endangered species *Alligator sinensis*, which is endemic to China. A total of 170 *BTB* genes were identified from the *A. sinensis* genome, classified into 13 families, and unevenly distributed across 16 chromosomes. Analysis of gene duplication events yielded eight pairs of tandem duplication genes and six pairs of segmental duplication genes. Phylogenetics shows that the *AsBTB* genes are evolutionarily conserved. The cis-regulatory elements in the *AsBTB* family promoter region reveal their involvement in multiple biological processes. Protein interaction network analysis indicates that the protein interactions of the *AsBTB* genes are centered around CLU-3, mainly participating in the regulation of biological processes through the ubiquitination pathway. The expression profile and protein interaction network analysis of *AsBTB* genes during sex differentiation and early gonadal development indicate that *AsBTB* genes are widely expressed in this process and involves numerous genes and pathways for regulation. This study provides a basis for further investigation of the role of the *BTB* gene in sex differentiation and gonadal development in *A. sinensis*.

## 1. Introduction

*BTB/POZ* (bric-a-brac/tramtrack/broad complex/poxvirus and zinc finger) gene superfamily is widely distributed in higher eukaryotes and is characterized by the presence of the BTB/POZ domain, a common protein–protein interaction motif of approximately 100 amino acids [1,2,3,4,5,6]. The BTB domain (also known as the POZ domain) was originally identified as present in *Drosophila*, transcriptional regulators, as well as many poxvirus zinc finger proteins [1,7,8,9]; the domain core consists of 5 α-helices—A1/2 and A4/5 form 2 α-helical hairpins and 3 β -strands form one β -sheets [6,10]. In addition to the BTB core domain, different BTB proteins also include the amino acid (N) terminus and the carboxyl (C) terminus BTB extension region [5,6,11,12,13,14], including the ZF (zinc finger), KELCH (Kelch repeat), MATH (meprin and TRAF-C homology) domain, ANK (ankyrin repeat), bZIP (basic leucine zipper), PHR (photolyase-homologous region), ATS1 (Alpha-tubulin suppressor ATS1, and related RCC1 domain-containing). These domains contribute to the functional diversity of protein families and are important for the wide range of molecular functions of the *BTB* gene [5]. The *BTB* genes were classified into different gene families based on additional domains in the structural extension region of the BTB protein, including BTB-ZF (BTB-zinc finger), BBK (BTB-BACK-Kelch), T1-Kv (voltage-gated potassium channel T1) [15], MATH-BTB, BTB-NPH3, and BBP (BTB-BACK-PHR) [6].

The *BTB* gene superfamily possesses a wide and complex array of functions. The oldest BTB domain is functionally related to an E3 ubiquitin ligase [16]. In higher phylogenetic populations, *BTB* genes undergo specific adaptations, acquire new functions, and play a role in transcription, chromatin remodeling, cytoskeleton dynamics, and ion channel formation [7,8,17,18,19,20,21]. This makes the *BTB* gene family crucial in a multitude of eukaryotic events, including development [5,22,23], tumorigenesis [24,25,26], and gametogenesis [27,28,29], among others. Recent research has found that the *BTB* gene family plays a very important role in the process of gender differentiation and gonadal development, mainly through two mechanisms (ubiquitination and transcription regulation). Firstly, members of the *BTB* gene superfamily are recruited by cullin-3 (Cul-3) proteins to E3 ubiquitin ligase complexes to participate in substrate recognition, selectively recruiting target proteins for ubiquitination [30]. Through this mechanism, the *BTB* superfamily genes play a role in the determination of the fate of primitive germ cells (PGC) [31,32,33], oogenesis [34], and spermatogenesis [35,36,37]. In *Drosophila*, the BBK protein germ cell-less (GCL) is a key regulatory factor in the formation of PGC [32,33]. As a substrate-specific adapter of the cullin3-RING ubiquitin ligase complex, GCL promotes the fate of PGC by mediating the degradation of the tyrosine kinase receptor (RTK) torso through the ubiquitination pathway [31]. In mammals, the *Gmcl1* homologous gene of *Gcl* in humans and mice is also closely related to spermatogenesis [38,39]. In *Drosophila*, the BBK protein Klhl-10 interacts with CUL-3 to form a CUL3-based ubiquitin ligase complex [36]. During spermatogenesis, the expression of Klhl-10 degrades caspase inhibitors through ubiquitination, activates effector caspase, and removes a large amount of cytoplasm during the maturation of sperm [40,41]. Studies have shown that Klhl-10 has the same mechanism in the process of mammalian spermatogenesis [35,42,43]. Secondly, studies have shown that many BTB proteins contain DNA-binding domains and function as transcriptional regulatory factors [6,44,45,46]. The main components of these are BTB-ZF proteins, also known as POK (POZ and Krüppel zinc finger) proteins [47]. Although many members of the *BTB* superfamily are associated with transcriptional repressors [44,48], other members have been found to function as transcriptional activators by interacting with co-activators or by mediating chromatin remodeling [49,50,51]. The *BTB* superfamily genes play a role in processes such as gonadogenesis [52,53], spermatogenesis [54,55,56], oogenesis [57,58,59,60], meiosis [28,61], and cell migration [62] through transcriptional regulation. In fruit flies, the BTB-ZF gene longitudinal lacking (*lola*) and the *slit/robo* pathway are essential for gonad morphogenesis [52,53]. *Lola* is necessary for the maintenance of germline stem cells (GSCs) and somatic cyst stem cells (CySCs), as well as germ cell transition from spermatogonia to spermatocytes [46]. In mammalian testes, the BTB-ZF protein PLZF inhibits the mammalian target of rapamycin complex 1 (mTORC-1) by inducing the expression of mTORC-1 inhibitory factor Redd-1, thereby promoting the self-renewal of spermatogonial stem cells [56,63,64]. Although research on the function of *BTB* genes and their role in gonadal development and sex differentiation has been conducted in some model species and mammals, yielding certain results, studies on amphibians, reptiles, and birds remain lacking.

The Chinese alligator (*Alligator sinensis*) is an endangered species endemic to China. It has been listed on the Red List of Threatened Species by the International Union for Conservation of Nature (IUCN). The *A. sinensis* is of high economic, ecological, and scientific value, but its wild population is on the verge of extinction, falling to less than 150 [65]. Studying the developmental and reproductive mechanism of *A. sinensis* has far-reaching significance for the restoration of its population size. In the past, we conducted extensive research on the development and reproductive mechanisms of *A. sinensis*, including processes such as egg incubation [66,67], early embryonic and organ development [68,69,70], oogenesis [71,72], and sex differentiation [73]. However, the study of many mechanisms remains insufficient. Unlike mammals and birds, *A. sinensis* lacks sex chromosomes, and its sex is determined by the incubation temperature of the egg during a specific period of development [74]. Although research on the temperature-dependent sex-determination mechanism of *A. sinensis* has begun [75], the molecular mechanisms of numerous key genes and transcriptional regulatory factors involved in temperature sex determination and subsequent early gonadal development remain obscure. Genome-wide studies of the *BTB* gene superfamily have been conducted in several species, including *Saccharomyces cerevisiae*, *Dictyostellium discoideum*, *Arabidopsis thaliana*, *Solanum lycopersicum*, *Oryza sativa*, *Caenorhabditis elegans*, *Bombyx mori*, *Drosophila melanogaster*, *Danio rerio*, *Takifugu rubripes*, *Ratus norvegicus*, *Mus musculus*, *Homo sapiens*, *Glycinemax (Linn.) Merr*, etc. [1,6,14,76,77,78]. However, there is currently no systematic report on the *BTB* gene superfamily of the *A. sinensis*. In birds and reptiles, there is also a lack of systematic research. At the same time, given the important role of the *BTB* gene superfamily in animal development and reproduction, studying the *BTB* gene superfamily of the *A. sinensis* and its expression patterns during sex differentiation and early gonadal development is of significant importance for elucidating the reproductive mechanisms of the *A. sinensis*.

This study uses available *A. sinensis* genome sequence data to conduct a comprehensive study of the *BTB* gene superfamily from the aspects of gene structure, motif composition, chromosome positioning, gene duplication events, phylogenetic relationships, cis-acting element composition, and protein interactions. In addition, we also analyzed the expression profile of the *BTB* gene in the process of gender differentiation and early gonadal development, as well as the protein regulatory network of differentially expressed genes. RT-qPCR validation was also performed on transcriptome sequencing results. The purpose of this study is to systematically analyze the sequence structure of the *A. sinensis BTB* gene superfamily, explore the evolutionary relationship of the *AsBTB* gene superfamily, reveal the expression and regulation of *AsBTB* gene superfamily members in early stages of sex differentiation and gonad development, and lay a foundation for further studying the function of the *A. sinensis BTB* gene superfamily.

## 2. Results

### 2.1. Identification, Classification, and Distribution of BTB Genes in the A. sinensis Genome

In this study, 170 *BTB* genes were identified in the *A. sinensis* genome and were renamed from *AsBTB1* to *AsBTB170*. The information for these *BTB* genes and their corresponding proteins is shown in Additional file S1: Table S1 and Additional file S2: Table S2, namely, the name, gene ID, affiliation chromosome, location on the chromosome, number of CDS regions, protein length (AA), molecular weight (MW), theoretical isoelectric point (pI), affiliation classification, and subcellular location. Protein length varies greatly from 189 aa (*AsBTB15*) to 1817 aa (*AsBTB15*). The molecular weight ranges from 18108.34 (*AsBTB140*) to 206207.72 Da (*AsBTB32*), and the theoretical isoelectric point (pI) ranges from 4.62 (*AsBTB15*) to 9.47 (*AsBTB59*). Subsequently, we classified the AsBTB gene based on search data from the Pfam database, SMART database, and NCBI CDD database, following the BTB gene classification method published by Peter in 2005 [6]. The results show that the *A. sinensis BTB* superfamily is divided into 13 gene families, as follows: 50 BBK, 44 BTB-ZF (only the BTB protein containing C2H2 zinc finger domain), 25 T1-KV, 23 BTBonly, 7 BTB-KCTD, 5 ANK-BTB-BACK, 4 RhoBTB, 3 BBP, 2 MATH-BTB, 2 BTB-bZIP, 2 BTB-BEN, 2 ATS1-BTB-BACK, and 1 BTB-FYVE (*AsBTB156*). Subcellular localization prediction results showed that 80 *AsBTB* members were located in the nucleus, 40 in the plasma membrane, 29 in the cytoplasm, 7 in the mitochondria, 6 in the extracellular matrix, 6 simultaneously in the cytoplasm and nucleus, and only 1 member of the *BTB* superfamily each in the cytoskeleton and peroxisomes.

**Figure 1:**
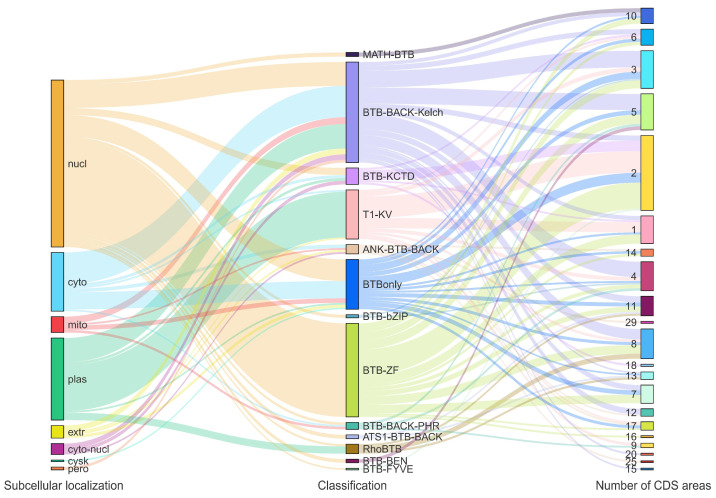
Sankey diagram of the correlation between *AsBTB* gene classification, subcellular localization, and number of CDS regions. Each rectangle represents a category and the number of members is visualized according to the length of the rectangle. Lines between rectangles represent the correlation between them.

The Sankey diagram was constructed based on the prediction of subcellular localization, classification, and the number of CDS regions of *A. sinensis BTB* gene superfamily members. As shown in Figure 1, BTB-ZF and T1-KV subcellular localization are conserved, with all BTB-ZF members localized to the nucleus and 23 of the 25 T1-KV members localized to the cytoplasmic membrane. BTB-BEN and MATH-BTB are conserved in subcellular localization and the number of CDS regions, and other *BTB* gene families show different degrees of diversity in these two dimensions. Overall, the number of CDS regions is more diverse in the different family classifications of *BTB* genes, but the predicted subcellular localization results are more conservative.

**Figure 2:**
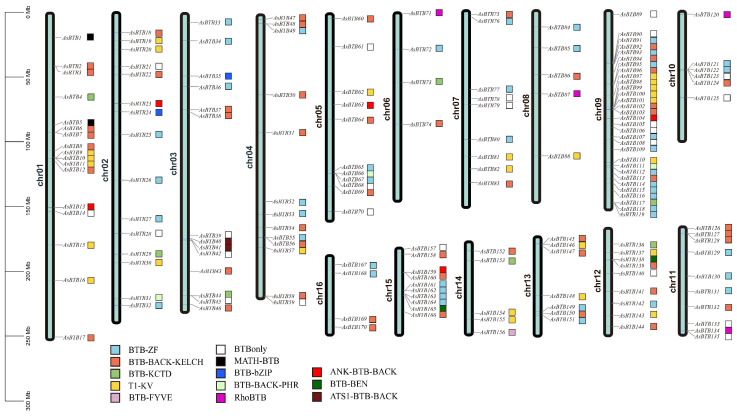
Distribution of *AsBTB* genes on the 16 chromosomes of *A. sinensis*. The scale bar on the left shows the length of the *A. sinensis* chromosome, and the box on the right of the gene name shows the classification of *AsBTB*.

**Figure 3:**
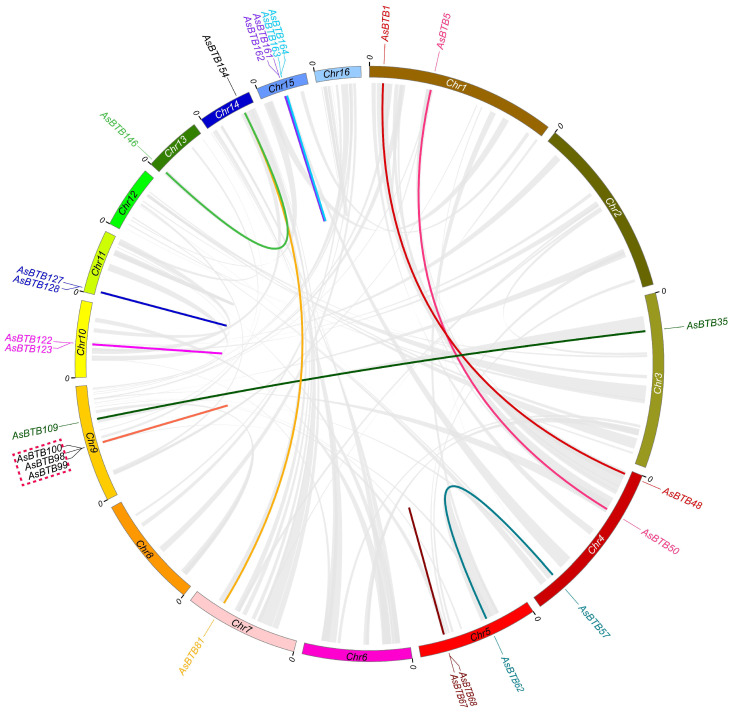
Repeat of the *AsBTB* genes on 16 chromosomes of the *A. sinensis* genome. Colorful lines connect tandem repeated gene pairs and segmental repeated gene pairs. The red dashed box indicates that the three genes are mutually tandem duplicated gene pairs. The corresponding relationships of duplicated genes are listed in Additional file S3: Table S3.

All 170 *AsBTB* genes were unevenly distributed across all 16 chromosomes of the *A. sinensis* (Figure 2). Chromosome 9 contains the most 31 *AsBTB* genes, chromosome 1 contains 17 *AsBTB* genes, and chromosome 2 has 15 *AsBTB* genes. In contrast, chromosomes 6 and 16 contain only 4 *AsBTB* genes each. In our study, eight pairs of tandem duplication genes and six pairs of segmental duplication genes were identified in the *A. sinensis AsBTB* gene family (Figure 3, Additional file S3: Table S3), indicating that both segmental duplication and tandem duplication events play a role in the amplification of the *AsBTB* gene superfamily.

### 2.2. Phylogenetic Analysis of the AsBTB

**Figure 4:**
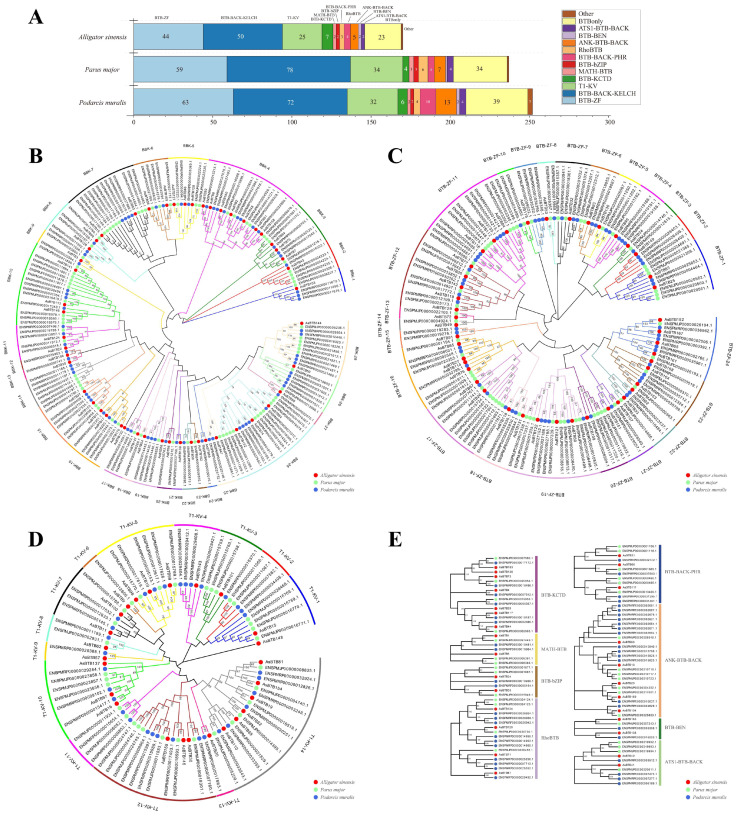
Identification, classification, and phylogenetic tree of *BTB* genes in *A. sinensis*, *P. major,* and *P. muralis*. (**A**) Identification and classification of *BTB* genes in the genomes of *A. sinensis*, *P. major,* and *P. muralis*; (**B**) Phylogenetic tree of the BBK genes; (**C**) phylogenetic tree of the BTB-ZF genes; (**D**) phylogenetic tree of the T1-KV genes; (**E**) phylogenetic tree of the other genes. The red circle labels the *A. sinensis* gene, the green circle labels the *P. major* gene, and the blue circle labels the *P. muralis* gene. Different colored boxes represent different *BTB* gene categories.

To identify the phylogenetic relationships of the *A. sinensis BTB* gene superfamily, we identified and classified the *BTB* gene families in the *Parus major* and *Podarcis muralis* genomes, respectively. The results are in Figure 4A and Additional file S4: Table S4; the total of 170 *BTB* gene families in *A. sinensis* is slightly less than that of *P. major* (238) and *P. muralis* (252), but the type and number of *BTB* gene families among the three are relatively conservative, with all sharing the same distribution of 12 BTB types.

Due to the large sequence difference of *BTB* genes with different domains [6], we generated phylogenetic trees separately using the MEGA11 maximum likelihood (ML) method based on the *BTB*-conserved domain protein sequences of BBK (Figure 4B), BTB-ZF (Figure 4C), T1-KV (Figure 4D) and the full-length protein sequences of other families (Figure 4E) (the BTBonly protein was removed due to its low homology). The BBK family was clustered into 29 subfamilies, the BTB-ZF family into 24 subfamilies, the T1-KV family into 14 subfamilies, and the rest of the classifications into one subfamily each, based on bootstrap values (>50%) of the phylogenetic tree. Among them, although the number of *A. sinensis BTB* genes is small, they are widely distributed (only missing four subfamilies (BBK-14, BTB-ZF-9, T1-KV-2, and T1-KV-11); *P. muralis* is lacking genes in five subfamilies (BBK-18, BBK-23, BTB-ZF-13, BTB-ZF-14, and T1-KV-6), and *P. major* is lacking genes in eight subfamilies (BBK-12, BBK-13, BBK-21, BBK-24, BTB-ZF-15, T1-KV-4, T1-KV-9, and T1-KV-13). These results suggested a common ancestor of the *BTB* gene among *A. sinensis*, *P. major,* and *P. muralis*, as well as respective specific amplification and loss during the evolution after isolation. *A. sinensis* is conserved in the evolution of this gene superfamily.

**Figure 5:**
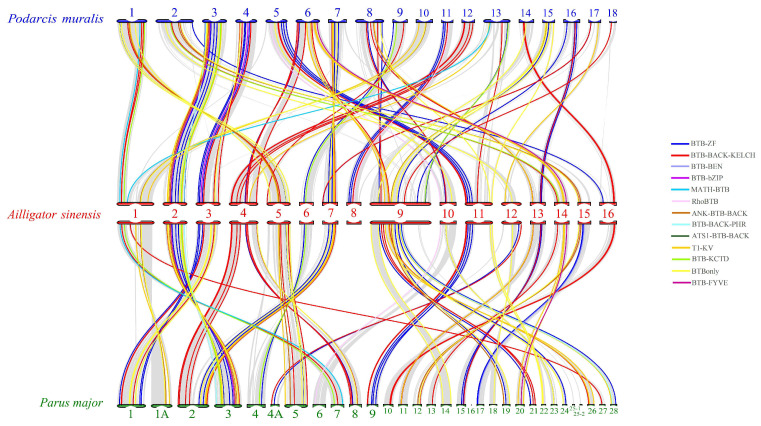
*A. sinensis*, *P. major,* and *P. muralis BTB* collinearity analysis. Numbers represent chromosome numbers. *P. muralis* chromosomes are shown in blue, *A. sinensis* in red, *P. major* in green. Non-BTB homologous genes are linked by gray lines and BTB homologous genes are linked by colored lines depending on the category.

Subsequently, we conducted a collinearity analysis between *A. sinensis, P. major,* and *P. muralis*, respectively (Figure 5 and Additional file S5: Table S5). The results showed that *A. sinensis* had 123 *BTB* homologous pairs with *P. muralis*, dispersed across all chromosomes, and 125 *BTB* homologous pairs with *P. major*, dispersed on all chromosomes, except chromosome 8. Among these, 98 gene pairs were jointly homologous among the 3. It is obvious that the *BTB* superfamily genes were conserved during evolution, implying that they may be functionally conserved.

### 2.3. Gene Structure, Protein Structure, and Protein Motif Analysis of AsBTB

**Figure 6:**
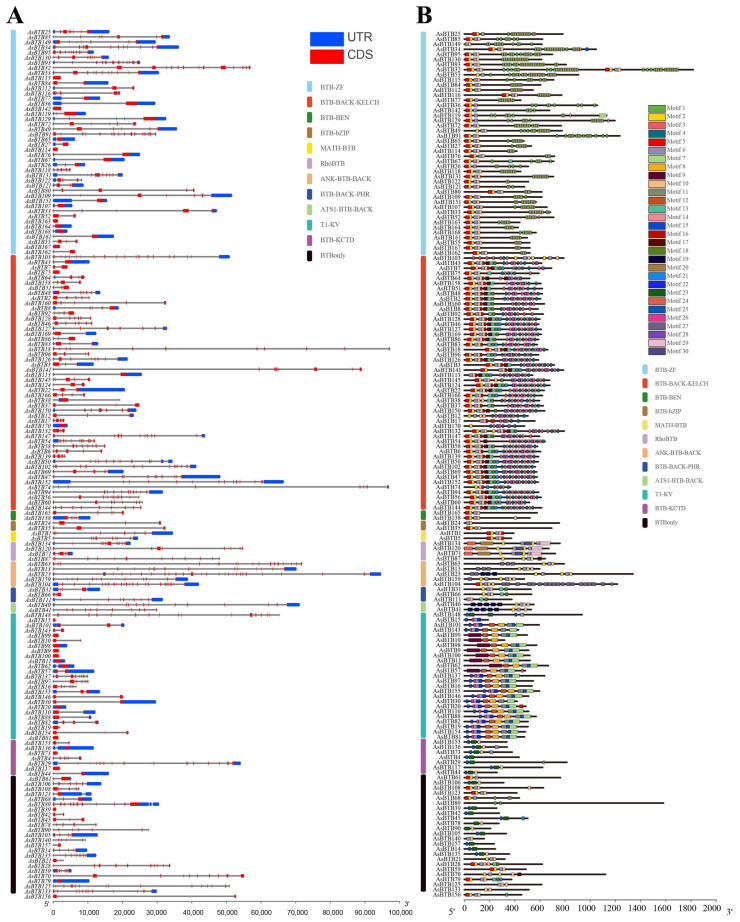
Gene structure and protein conserved motifs of *A. sinensis*. (**A**) Gene CDS-UTR structure of AsBTBs. Blue: UTR, red: CDS, spaces between the boxes: introns. The scale bar at the bottom indicates the length of gene. Colored lines on the left side of the gene name represent the category to which the gene belongs. (**B**) The conserved motif of AsBTB proteins. Different motifs are shown with different colored boxes and numbers (1–30). The gray lines represent the non-conserved sequences. The lengths of motifs can be estimated using the scale at the bottom.

To better explore the relationship between the genes and functions of the *A. sinensis BTB* superfamily, we analyzed their exon/intron structures and conserved motifs. First, we mapped their gene structures, including the CDS and UTR regions (Figure 6A). The 170 *AsBTB* genes contain between 1 and 29 CDS regions, which vary widely. Some *AsBTB* genes contain a large number of CDS regions, such as *AsBTB23* (29 CDS regions), *AsBTB103* (20 CDS regions), and *AsBTB104* (25 CDS regions). Conservation of CDS regions was only seen in individual families with fewer members, such as MATH-BTB (10 CDS regions). To further analyze the evolution of AsBTB proteins, we used MEME to identify 30 conserved motifs of the 170 members of this protein family. The length and conserved sequences of each motif are listed in the Additional file S6: Table S6. As expected, all AsBTB proteins in the same family have similar motif compositions except BTBonly, despite the large difference in the number of introns (Figure 6B), indicating that the motif analysis supports the classification of the *AsBTB* family, which also means that there may be similar functions.

**Figure 7:**
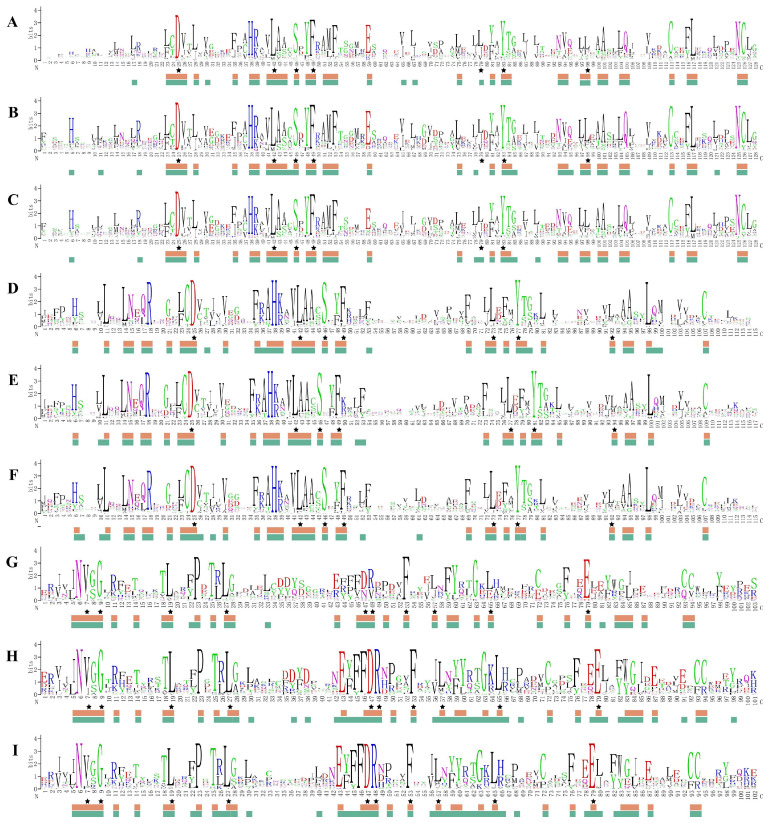
Sequence logos of the BTB structural domain in *A. sinensis*, *P. major,* and *P. muralis*. Sequence logos of the BTB structural domain in the BBK genes (*A. sinensis* (**A**), *P. major* (**B**), *P. muralis* (**C**)), BTB-ZF genes (*A. sinensis* (**D**), *P. major* (**E**), *P. muralis* (**F**)) and T1-KV genes (*A. sinensis* (**G**), *P. major* (**H**), and *P. muralis* (**I**)). Amino acid residues marked with asterisks are conserved (probability of more than 50%) in the BTB structural domains of all organisms (data are referenced from the Pfam database https://www.ebi.ac.uk/interpro/entry/pfam/#table, accessed on 1 October 2023.). Amino acid residues conserved in the three species of *A. sinensis*, *P. major,* and *P. muralis* are marked with brown squares. Amino acid residues conserved in single species are marked in green squares.

To further explore the conserved structural domains of AsBTB proteins, multiple sequence comparisons of the amino acid sequences of the BTB structural domains in the BBK, BTB-ZF, and T1-Kv gene families were performed in *A. sinensis*, *P. major*, and *P. muralis*, respectively. Sequence identities were generated by WebLogo (Figure 7), where amino acid residues exceeding 50% occurrence probability were annotated and the amino acid site was considered conserved. The results showed that there were 36, 34, and 33 conserved amino acid residues in the BTB domain of BBK protein, BTB-ZF protein, and T1-Kv protein in the three species, respectively. In the conserved domain sequence of BTB in a single species, there were 41, 45, and 42 conserved amino acid residues of the BBK protein in *A. sinensis* (Figure 7A), *P. major* (Figure 7B), and *P. muralis* (Figure 7C), respectively. In the conserved BTB domain of the BTB-ZF protein, there were 40, 37, and 40 conserved amino acid residues in *A. sinensis* (Figure 7D), *P. major* (Figure 7E), and *P. muralis* (Figure 7F). In the conserved BTB domain of the T1-Kv protein, there were 36, 52, and 49 conserved amino acid residues in *A. sinensis* (Figure 7G), *P. major* (Figure 7H), and *P. muralis* (Figure 7I). All BTB members in these three taxa have typical features of BTB-conserved structural domains. It is evident that the BTB-conserved domain sequences exhibit significant variation across different families, with relatively minor variations within each family. This suggests that BTB superfamily genes may have relatively conserved functions within the same family.

### 2.4. Analysis of the Cis-Acting Elements in the Promoter Region of the AsBTB Gene

**Figure 8:**
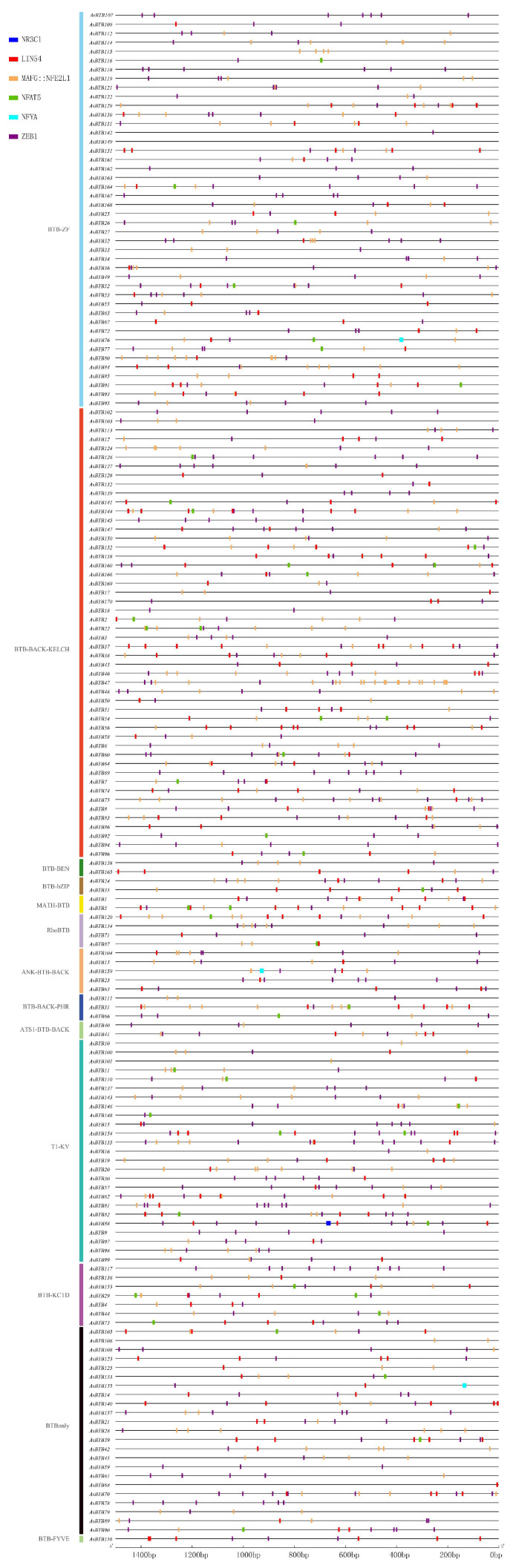
Distribution of cis-acting elements in the promoter region of *AsBTB* genes. The cis-acting elements of the *AsBTB* gene promoter region (1500bp upstream of the start codon) were identified using JASPAR (an online program). Different shapes and colors represent the different types of cis-elements. The statistics of the cis-elements are listed in the Additional file S7: Table S7.

To understand the regulatory patterns of transcription and expression of *AsBTB* genes, we analyzed their promoter cis-acting elements. We used JASPAR to identify cis-acting elements in the 1.5 kb region upstream of the *AsBTB* translation start site using all transcription factors of *Gallus gallus* (Additional File S7: Table S7). As shown in Figure 8, In total, the six cis-element binding sites were identified, LIN-54 (lin-54 DREAM MuvB core complex component), NFAT-5 (nuclear factor of activated T cells 5), NR3C-1 (nuclear receptor subfamily 3 group C member 1), ZEB-1(zinc finger E-box binding homeobox 1), MAFG::NFE2L-1 (MAF bZIP transcription factor G and NFE2 like bZIP transcription factor 1), and NFYA (nuclear transcription factor Y subunit alpha). Of these, 304 LIN-54 binding sites and 463 ZEB-1 binding sites were widely distributed in all 13 *AsBTB* family classifications; 328 MAFG::NFE2L-1 binding sites were present in 12 taxa (except for BTB-FYVE), and 46 NFAT-5 binding sites were present in 10 *AsBTB* family taxa (except ATS1-BTB-BACK, BTB-BEN, and ANK-BTB-BACK). The three NFYA binding sites were located in BTBonly, ANK-BTB-BACK, and BTB-ZF, and the NR3C-1 binding site was only in T1-KV. The cis-acting elements in the promoter region of *AsBTB* genes are mainly involved in cell cycle regulation, embryonic development, cell differentiation, transcription regulation, oxidative stress, and ubiquitination regulation, and partially involved in the immune and inflammatory responses and glucocorticoid regulation of hypertonic stress. Although there are differences in the abundance and distribution of NFYA and NR3C-1, overall, the widespread distribution of LIN-54, NFAT-5, ZEB-1, and MAFG::NFE2L-1 in different *AsBTB* gene promoter regions reflects the conservation of cis-acting elements in the *AsBTB* promoter region, suggesting the possible correlation of the members of this gene family in transcription regulation and biological processes.

### 2.5. Protein Interaction Network Analysis of the AsBTB Genes

**Figure 9:**
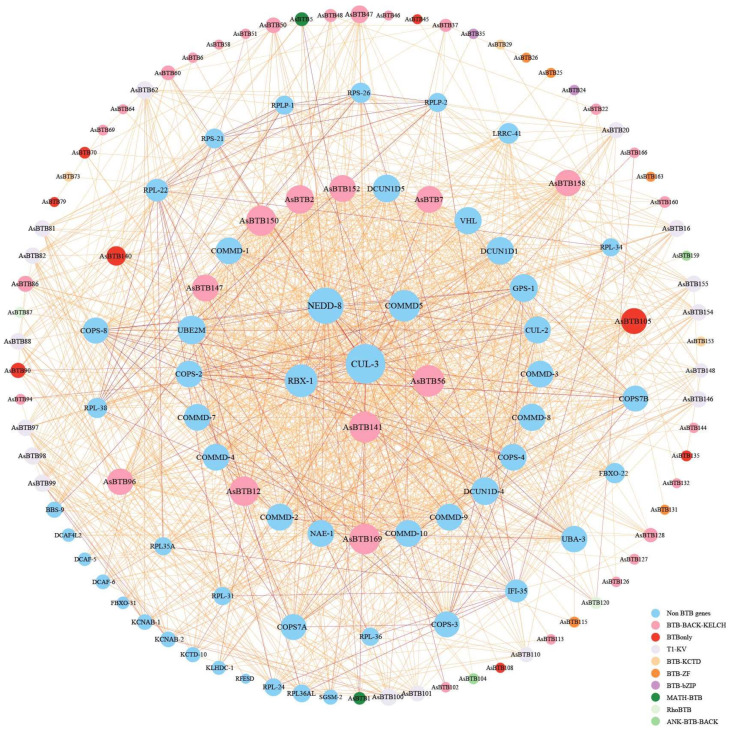
Protein interaction network of *AsBTB*. The *AsBTB* type is visualized by the circle color, where blue represents non-BTB functional proteins. The circle size corresponds to the number of interacting genes and the depth of the lines represents the magnitude of confidence. All protein interaction relationships have a confidence score that is greater than or equal to 0.7.

Chicken, serving as a model species closely related to crocodilians, holds significant reference value for studying the functions of *A. sinensis* genes. To elucidate the mechanism of interaction between AsBTB proteins, we predicted their protein–protein interactions based on homologous BTB proteins in *Gallus gallus*. Homologous similarity results are presented in Additional file S8: Table S8, protein interaction network confidence ≥ 0.7. The results show (Figure 9) that there are 78 AsBTB proteins (34 BBK, 16 T1-Kv, 11 BTBonly, 6 BTB-ZF, 3 BTB-KCTD, 2 RhoBTB, 2 MATH-BTB, 2 ANK-BTB-BACK, 2 BTB-bZIP) and 53 functional proteins that interact with them. With CUL-3 as the center (with 70 protein interactions), the protein interaction network is represented by four concentric circles (from the inside to the outside, the first ring includes 69-50 protein interaction, the second ring includes 49-40 protein interactions, the third ring includes 39-20 protein interactions, and the fourth ring includes 19-1 protein interactions). It can be seen that in the *AsBTB* family, only BBK family members are in the second ring of the transcriptional regulatory network; two BTBonly and two BBK are located in the third ring; and the remaining *AsBTB* type members are on the outermost periphery. Interestingly, the number of predicted BTB-ZF member protein interactions is less than or equal to 3. The CUL proteins, including CUL-3 and CUL-2, are important in this network and play a key role in ubiquitination. Moreover, the COMMD family is distributed in the internal ring (including COMMD-5, COMMD-7, COMMD-4, etc.), and contributes to many dispersed biological functions through the ubiquitination pathway. NRDD-8 and RBX-1, located on the first loop of this network, regulate ubiquitin ligase activity as well as cell cycle regulation through ubiquitination processes, respectively. DCUN1D-1, DCUN1D-3, DCUN1D-4, and other genes are located in the second loop, participating in the positive regulation of protein neddylation and the regulation of protein ubiquitination, enabling the protein-binding activity of cullin family proteins. On the third and fourth rings, COPS-2, COPS-4, and COPS-8 act as positive regulators of CUL, regulate different signaling pathways through the ubiquitination process. Ribosomal proteins, such as RPL-38 and RPS-26, regulate transcription, KCNAB-1, and KCNAB-2 ion channel proteins. In conclusion, *AsBTB* genes, as important components of the bio-ubiquitination process, with CLU-3 at the core, enable many scattered signaling pathways, biological processes, and functions to be regulated through the ubiquitination pathway.

### 2.6. Expression Pattern and Protein Network Analysis of AsBTB Genes during Sex Differentiation and Early Gonadal Development in Alligators sinensis

**Figure 10:**
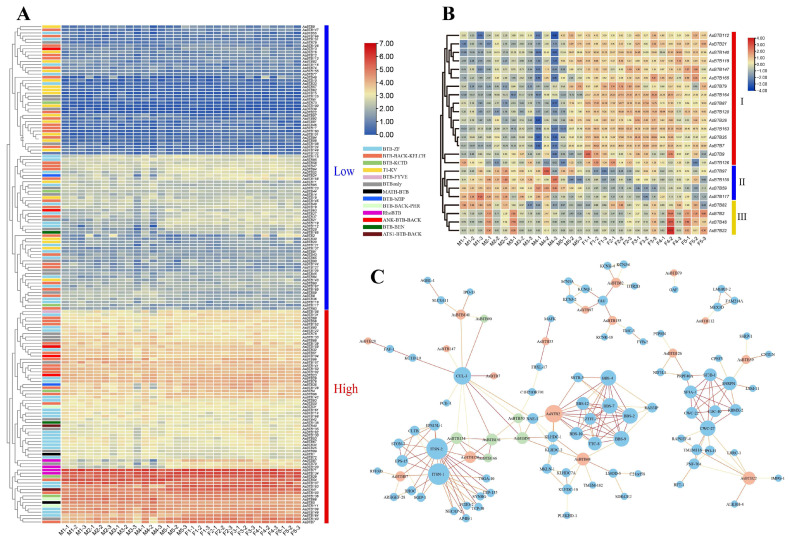
Expressions and protein interactions of *AsBTB* genes during the sex differentiation process and early gonadal development in *A. sinensis*. (**A**) *AsBTB* gene expression in the sex differentiation and early gonadal development process of *A. sinensis*. Red and blue indicate differences in expression levels in each sample, respectively, and the different colored rectangles on the left side of the expression profile visualize the *AsBTB* gene types. (**B**) Expression profiles of *AsBTB* differentially expressed genes during the sex differentiation and early gonadal development process in *A. sinensis*. FPKM values are displayed in the box, and the FPKM values are visualized after the log10 transformation. (**C**) Differentially expressed *AsBTB* gene protein interactions during the sex differentiation and early gonadal development process in *A. sinensis*. Blue circles indicate non-BTB genes, red circles indicate differentially expressed *AsBTB* genes, and green circles indicate *AsBTB* genes with differential expression. Circle sizes denote the number of interacting genes, and the depth of the lines represents the magnitude of confidence. All protein interaction relationships have a confidence score that is greater than or equal 0.7.

In order to analyze the expression pattern and protein regulatory network of *AsBTB* genes during sex differentiation and early gonadal development in *Alligators sinensis*, we extracted the expression levels of the *AsBTB* genes at various stages of sex differentiation based on transcriptome data and presented them (Figure 10A and Additional file S9: Table S9). Genes with no or low expression levels were excluded (FPKM < 0.5). Subsequently, genes with padj < 0.05 and log2foldchange ≥ 1 were screened as differentially expressed genes (DEGs) (results are shown in Figure 10B). Finally, based on the homologous BTB protein in the chicken, we predicted the protein–protein interactions of DEGs, as shown in Figure 10C.

A total of 157 expression profiles of *AsBTB* genes were obtained and divided into two expression patterns: low-expression genes (90 genes) and high-expression genes (67 genes) (Figure 10A). Some *AsBTB* gene families were distributed in both low and high expressions, such as 45 BBK genes (30 low-expression and 15 high-expression), 43 BTB-ZF genes (20 low-expression and 23 high-expression), 22 BTBonly genes (13 low-expression and 9 high-expression), 6 BTB-KCTD genes (3 low-expression and 3 high-expression), 5 Ank-BTB-BACK genes (2 low-expression and 3 high-expression), 2 BTB-BEN genes (1 low-expression and 1 high-expression), and 2 BTB-bZIP genes (1 low-expression and 1 high-expression). At the same time, another class of *AsBTB* gene superfamily members was only exhibited in one of the high or low expression patterns; all 20 T1-Kv genes were lowly expressed; and four RhoBTB genes, three BTB-BACK-PHR genes, two ATS1-BTB-BACK genes, two MATH-BTB genes, and one BTB-FYVE gene were highly expressed.

In this process, 24 *AsBTB* genes were DEGs (Figure 10B). In the embryonic gonads of different genders, they were divided into three groups according to different expression patterns. The first group consisted of 16 *AsBTB* genes, which had significantly higher expression in female gonads than in male gonads. The second group consists of four *AsBTB* genes with significantly lower expression in the female gonads than in the male gonads. The third group consisted of four *AsBTB* genes, and the expression differences could not be classified according to sex. In the differential analysis of adjacent periods of the same sex, *AsBTB21*, *AsBTB119*, *AsBTB87,* and *AsBTB28* were significantly upregulated during the M5 period, and *AsBTB155*, *AsBTB2,* and *AsBTB48* were significantly downregulated during this period. *AsBTB9* was significantly downregulated in the F2 period, *AsBTB97* was significantly upregulated in the F3 period, and *AsBTB22* and *AsBTB2* were significantly upregulated in the M2 period.

In addition, *AsBTB2* was also significantly upregulated during the F3 period. Based on the predicted protein interactions of the homologous BTB proteins in *Gallus gallus*, as shown in Figure 10C, a protein interaction network of 17 genes, scattered in five clusters, was identified. Meanwhile, we found 90 corresponding interacting function genes, of which, 6 were also *AsBTB* genes. Protein interactions involve the key genes CUL-3, ITSN-1, ITSN-2, BBS-4, BBS-7, BBS-2, BBS-9, CWC-27, FAU, etc. The expression patterns and functions of *BTB* members during the sex differentiation and early gonadal development showed considerable differentiation and involved numerous genes and biological processes.

**Figure 11:**
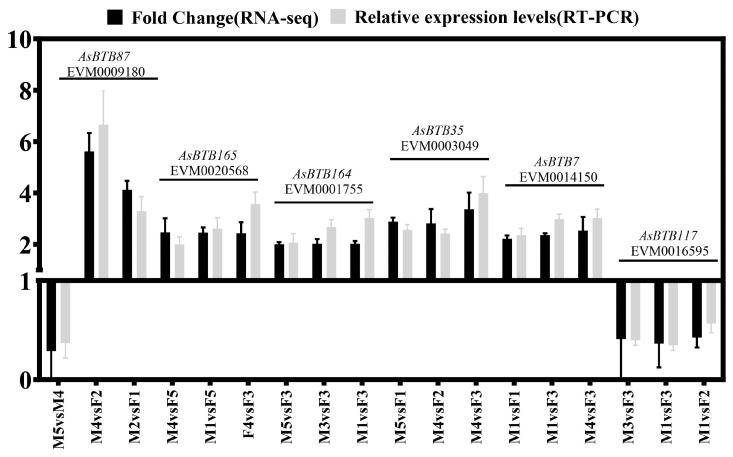
Expression profiles of six *AsBTB* genes during the sex differentiation process and early gonadal development in *A. sinensis*. The area above the Y = 1 baseline represents the upregulation of genes, while the area below it represents the downregulation.

To verify the accuracy of transcriptome data, we randomly selected six genes, namely, *AsBTB87*, *AsBTB165*, *AsBTB164*, *AsBTB35*, *AsBTB7*, and *AsBTB117*, for RT-qPCR experiments. We used the β-actin gene of *A. sinensis* as the reference gene. The RT-qPCR results were compared with the transcriptome data (Figure 11). Primer information is listed in Additional file S10: Table S10. As seen from the results, the expression patterns of all six AsBTB genes were consistent with the transcriptome data, validating the reliability of the transcriptome data outcomes.

## 3. Discussion

Members of the *BTB* gene family are widely distributed in eukaryotes. With the development of genome-sequencing technologies, genome-wide analysis of the *BTB* gene superfamily has been demonstrated in many species. This includes *Saccharomyces cerevisiae* (6), *Dictyostellium discoideum* (41), *Arabidopsis thaliana* (78), *Solanum lycopersicum* (38), *Oryza sativa* (110), *Caenorhabditis elegans* (181), *Bombyx mori* (56), *Drosophila melanogaster* (85), *Danio rerio* (207), *Takifugu rubripes* (179), *Ratus norvegicus* (191), *Mus musculus* (194), *Homo sapiens* (183), etc. [1,6,14,76,77]. In this study, a total of 170 *BTB* genes of *A. sinensis*, 238 of *P. major*, and 252 of *P. muralis* were identified (Figure 4A and Additional file S4: Table S4), which were slightly different from mammals and fish. Among them, the genome sizes of *A. sinensis*, *P. major,* and *P. muralis* were 2.3 Gb, 1 Gb, and 1.5 Gb, respectively [15,79,80]. In this case, there was no direct correlation between the number of *BTB* genes and genome sizes in vertebrates. According to the report, the distribution and quantity of *BTB* genes in different species were different. The number of BTB domain proteins—including BTB-ZF, BBK, and T1-Kv family members—in the genomes of mammals and fish was between 25 and 50, with the sum of other kinds of BTB proteins ranging between 40 and 50 [6]. In our study, this is basically consistent with the above hypothesis, but with slight differences. *P. major* had 72 BBK genes, 59 had BTB-ZF genes, *P. muralis* had 78 BBK genes and 63 BTB-ZF genes, which are slightly higher than this hypothesis. This is the direct cause of the quantitative difference in *BTB* genes. Combined with phylogenetic analysis, it can be seen that in BBK (Figure 4B) and BTB-ZF (Figure 4C), more subfamily members belong to *P. major* or *P. muralis* genes. For example, 3 of 5 BBK-1 genes belong to *P. muralis* and 4 of 6 BBK-2 genes belong to *P. major*. Also, the *A. sinensis* is more conserved in these subfamilies, usually only one to two. This suggests that the differences in *BTB* gene numbers between species are due to lineage-specific amplification and contraction. After further expansion of BBK and BTB-ZF in reptiles and birds, it can be anticipated that *BTB* genes may play more of a role in the ubiquitination process and transcriptional regulation of transcription. But in *A. sinensis*, these genes are widely distributed and conserved, may retain their original function, and are a good model species to study the function and evolution of *BTB* genes. The BTB domain originated in the early stages of eukaryotic evolution and was further specialized in multicellular organisms, reaching the highest diversity in vertebrates and higher plants [81]. We identified a total of 13 *BTB* families in *A. sinensis*. In *P. major*, we identified 12 *BTB* gene families, and in *P. muralis*, we identified 15 *BTB* gene families (Additional file S4: Table S4). It can be inferred that the *BTB* gene superfamily has most likely undergone domain shuffling and lineage-specific expansion (LSE). LSE is recognized as one of the main mechanisms of eukaryotic adaptation and the production of novel protein functions; it is frequently present in proteins involved in cell differentiation and the development of multicellular organisms [82]. For example, in vertebrates, BTB-ZF proteins play important roles in development and tissue differentiation and undergo LSE [12]. Meanwhile, gene family differentiation depends largely on changes in gene structure and accompanying changes in protein sequences and functions.

The *BTB* gene superfamily members have highly diverse characteristics in some aspects, but they also have certain conservativeness in other aspects, which leads to the abnormal complexity of the *BTB* gene superfamily. We found that the CDS region counts of *AsBTB* varied greatly, ranging from 1 to 29 (Figure 1), and that this diversification of the gene structure is similar to *BTB* genes in *Oryza rufipogon* and *Paulownia fortunei* [76,83]. The variation in the nucleotide sequence length among the 170 *AsBTB* genes reflects the complexity of *BTB* genes in the genome of *A. sinensis* (Additional file S2: Table S2). In addition, overall, the subcellular localization of the BTB protein is diverse and widely distributed in many subcellular locations (from the nucleus to the cell membrane). However, in the same type of *BTB* gene, the subcellular localization is relatively conserved, suggesting that the functions of the same type of *BTB* gene are correlated. The difference in molecular weight and isoelectric point values of the BTB protein among different family members indirectly indicates the variation of its function. Meanwhile, the AsBTB protein contains 30 different conserved motifs (Figure 6) but has the same type of composition and a similar quantity in the same family, which also indicates this complex characteristic. The above results are also similar in the *BTB* gene family of *Bombyx mori* [71]. Therefore, these structural differences and diversities emphasize that the evolutionary patterns between different families are complex and diverse. Although *BTB* genes have some conserved sequences in common, they also have abundant functions and are widely involved in many biological processes.

The cis-element of the promoter plays a key role in initiating gene expression. Genes containing different cis-regulatory elements in gene promoter sequences may lead to different expression patterns [84]. In our study, a total of six cis-element-related sequences were identified in the promoter region of the *BTB* gene. Among them, LIN-54, ZEB-1, MAFG::NFE2L-1, and NFAT-5 are widely present in the *AsBTB* genes and are involved in cell cycle regulation [85,86], cell differentiation, embryonic development [87,88], transcriptional regulation [89], and ubiquitination, regulating the immune and inflammatory response of hypertonic stress [90,91]. NR3C-1 and NFYA are rarely distributed in the *AsBTB* genes, and involve processes such as cell proliferation [92] and the regulation of glucocorticoids [93]. The wide distribution of certain cis-acting elements in the promoter region of the *BTB* gene, as well as the conserved components, may reflect differentiation and functional significance at the transcriptional level, as well as relevance in biological processes.

Many cellular processes are regulated by the degradation of some key proteins by the ubiquitin system [94]. The regulation of the ubiquitin system specificity is a widespread mechanism for BTB proteins, which act as adaptor molecules within the ubiquitin ligase complex E3 [30]. Previous studies on *Caenorhabditis elegans* [95] and human cells [96,97] have found that many different types of BTB proteins can bind to Cul3 as adaptor proteins, including BBK, ANK-BTB-BACK, MATH-BTB, and BTB-ZF [11]. In our results, a similar phenomenon was found, as shown in Figure 9. CUL-3 functions as a scaffold protein in the ubiquitin E3 ligase complex and interacts with 70 proteins in the BTB protein interaction network, including BTBonly, BBK, RhoBTB, MATH-BTB, and BTB-KCTD. This result supports the notion that BTB proteins have at least one widely shared function—binding to CUL proteins to carry out biological functions through the ubiquitination pathway [98,99]. Interestingly, BTB-ZF, as the second most abundant *BTB* gene family of *AsBTB*, and a transcriptional regulator, predicts only five *AsBTB* genes involved in protein interactions, with each BTB-ZF interacting with only 1–3 proteins; these interactions are less involved in ubiquitination-related protein processes. This implies that the *BTB* gene has undergone significant divergence over the long evolutionary process and may play an important role in biological processes other than ubiquitination.

The *BTB* gene has been proven to be involved in the processes of sex differentiation and gonadal development, involving various *BTB* genes, including BBK, BTB-ZF, BTBonly proteins, etc. [35,37,42,100,101,102,103,104,105]. In our results, eight gene families were differentially expressed during the stages of sex differentiation and early gonadal development (Figure 10B), indicating that a wide variety of different types of *AsBTB* genes are extensively involved in this process. Our RT-qPCR results also confirmed differential expression of *AsBTB* genes in the stages of sex differentiation and early gonadal development. It is noteworthy that five BTB-ZF genes are differentially expressed, considering that BTB-ZF plays an important role as a transcriptional regulatory factor in organ development and gametogenesis. Even with fewer protein interaction relationships, their functions during gonadal development are still worthy of attention. By analyzing the protein interaction networks of differentially expressed genes (Figure 10C), we found that the protein interaction network of 17 key *AsBTB* genes is distributed across five clusters, and the expression patterns of *AsBTB* gene members during the early stages of sex differentiation and gonadal development are different. It is speculated that this gene family may undergo functional divergence. Among them, it is noteworthy that four RhoBTB gene family genes have similar expression patterns, two of which (*AsBTB87* and *AsBTB120*) belong to differentially expression genes and are significantly differentially expressed during sex determination and early gonadal development of female *A. sinensis*. It has been reported that the RhoBTB gene binds to CUL 3 as an adaptor protein, playing an important role in the cell cycle, cell apoptosis, and vesicle trafficking [106,107,108]. Our prediction result also supports this view, as shown in Figure 10C, where the RhoBTB genes *AsBTB134* and *AsBTB120* can interact with CUL3. This may regulate the differential expressions of *AsBTB87* and *AsBTB120* in the gonads of female alligators, affect ITSN1/2 and other key genes involved in cell exocytosis and endocytosis [109], and regulate the biological processes in sex determination and early gonadal development of female alligators. The determination of specific functions still needs to be verified by experiments. Our results provide useful information for further functional exploration of genes involved in sex determination and early gonadal development of *A. sinensis*.

## 4. Materials and Methods

### 4.1. Identification of the BTB Protein Superfamily in A. sinensis

We screened candidate genes accurately by searching for the conserved BTB domain of the corresponding proteins. The BTB protein hidden Markov models (HMMs) PF00096 and PF02214 were downloaded from the Pfam website (https://www.ebi.ac.uk/interpro/entry/pfam) accessed on 1 July 2023 [110]. Using the results of previous laboratory studies, we obtained the genomic information of *A. sinensis* (unpublished) [111]. The target genes were searched using HMMER V3.3.2 software with a threshold E-value < 0.01. Duplicated genes were manually removed from the search results. Then we identified and retained genes with intact BTB domains as candidate genes through the SMART database (http://smart.embl.de/, accessed on 10 July 2023) [16], the NCBI CDD database (https://www.ncbi.nlm.nih.gov/cdd/, accessed on 10 July 2023), and Pfam database, accessed on 10 July 2023 [112], with a threshold of 1e-5. According to the different gene positions on the chromosome, we renamed the candidate genes as *AsBTB* and classified them based on other domains of the candidate genes [6]. In addition to *A. sinensis*, we also used the same method to conduct whole-genome identification and classification of the *BTB* genes of *P. muralis* and *P. major*. Genomic information for *P. muralis* and *P. major* was downloaded from Ensembl (https://asia.ensembl.org/, accessed on 20 July 2023). The molecular weights (Mws) and isoelectric points (pIs) of *AsBTB* were analyzed by using the online ExPASy tool (http://Web.ExPASY.Org/protparam/, accessed on 22 July 2023) [113]. Subcellular localization of the AsBTB protein was analyzed using online WOLF PSORT (https://wolfpsort.hgc.jp/, accessed on 25 July 2023) [114].

### 4.2. Chromosomal Localization and Gene Duplication

The chromosome location information of *BTB* genes was extracted from the *A. sinensis* genome annotation file, and all *BTB* genes were mapped to the *A. sinensis* chromosomes using Circos software v0.69-9 [115]. Gene duplication events were analyzed using the Multiple Collinearity Scan toolkit (MCScanX python-version) with default parameters [116]. Graphical display of chromosomal localization and replication events of the *BTB* gene by TBtools software v2.121 [117].

### 4.3. Phylogenetic Analysis and Gene Collinearity Analysis

To explore the evolutionary relationships of the *BTB* gene superfamily, a phylogenetic tree was constructed using BTB proteins with the same domain classifications in *A. sinensis*, *P. muralis,* and *P. major*. Multiple sequence alignment was performed using the ClustalW program in MEGA 11 v11.0.13 [118]. Due to the large sequence differences of the *BTB* gene superfamily members, the evolutionary tree cannot be constructed together [6]. Here, a phylogenetic tree was constructed using the maximum likelihood (ML) method with 1000 bootstraps, separately for different *BTB* gene families. The online tool iTOL (https://itol.embl.de/, accessed on 30 July 2023) showed the phylogenetic tree [119]. To show the collinearity of *BTB* genes from *A. sinensis* and other selected species, BTB protein sequences from *A. sinensis*, *P. muralis,* and *P. major* were first aligned to themselves using BLASTp, using a threshold of an e-value < 1 × 10^−7^ along with default parameters. MCScanX python-version was then used to detect the collinearity between species. Finally, the collinearity analysis map was constructed using TBtools software v2.121 [120].

### 4.4. Analysis of Gene Structure and Conserved Motifs

The target gene annotation was extracted from the *A. sinensis* transcriptome using the GSDS website (http://gsds.gao-lab.org/, accessed on 1 September 2023), which showed the CDS and UTR regions of the target protein [121]. The motif analysis was performed using the MEME server (http://meme-suite.org/tools/meme/, accessed on 1 September 2023) [122]. To conduct the protein structure analysis of the superfamily members, the setting parameters were as follows: the maximum number of motifs was set to 30, the optimum motif width was set from 6 to 200, and the other parameters were the system default parameters. The sequences of the conserved BTB domain in the BTB-ZF, BBK, and T1-Kv families of *A. sinensis*, *P. muralis,* and *P. major* were subsequently visualized using the WebLogo platform (http://weblo go.berkeley.edu/, accessed on 20 September 2023) [123].

### 4.5. Analysis of Cis-Acting Elements and Protein Interaction Networks in Promoter Regions

In the JASPAR online program (https://jaspar.elixir.no/, accessed on 15 October 2023), the sequences of the *AsBTB* promoter region (1500 bp upstream of the start codon) were searched against all the transcriptional regulators of *Gallus gallus*, with a relative profile score threshold of 95%, and the results were displayed using the GSDS website (http://gsds.gao-lab.org/, accessed on 18 October 2023). Homologous gene pairs between *A. sinensis* and *Gallus gallus* were identified using the OrthoVenn3 tool (https://orthovenn3.bioinfotoolkits.net/, accessed on 20 October 2023). Subsequently, based on the homologous genes of *A. sinensis* and *Gallus gallus*, the protein–protein interaction network was predicted using the STRING database (http://string-db.org/cgi/, accessed on 22 October 2023), with a confidence parameter set to 0.7, and displayed using Cytoscape v3.7.0 software [124].

### 4.6. AsBTB Gene Expression Analysis

In order to understand the expression patterns of *BTB* superfamily genes in *A. sinensis,* sex differentiation, and early gonadal development, we chose the previous transcriptome sequencing results from our laboratory as raw data (unpublished) [125]. In this study, male alligators were hatched at 33 °C and females at 29 °C. Considering different temperatures, the development progression was different. We selected the gonadal tissues of male *A. sinensis* on the 19th day (M1), 22nd day (M2), 25th day (M3), 28th day (M4), and 33rd day (M5) after hatching, as well as the gonadal tissues of female on the 22nd day (F1), 25th day (F2), 28th day (F3), 36th day (F4), and 44th day (F5) after hatching for RNA-seq sequencing. The transcript abundance of *A. sinensis BTB* genes was calculated as fragments per kilobase of exon model per million mapped reads (FPKM). *AsBTB* genes with no or low expression levels (FPKM < 0.5) were excluded. Heatmaps of the obtained gene expression patterns were created using TBtools software v2.121 [117] Genes with padj ≤ 0.05 and |log2(fold change)| > 1 were considered differentially expressed genes (DEGs).

### 4.7. Real-Time Fluorescence Quantitative PCR

We randomly selected genes *AsBTB87*, *AsBTB165*, *AsBTB164*, *AsBTB35*, *AsBTB7*, and *AsBTB117* for transcriptome sequencing validation through RT-qPCR. We extracted total RNA from the sample using TRIzol reagent (Gibco BRL, Massachusetts, USA). cDNA synthesis was performed using the PrimeScript™ RT reagent Kit with a gDNA Eraser kit (Takara, Beijing, China). The samples were analyzed using quantitative primer real-time fluorescence quantitative PCR (RT-qPCR). We used the *β-actin* gene of *A. sinensis* as the reference gene. There were three technical duplicates of each sample during RT-qPCR. Finally, the relative expression level was calculated via the 2^−ΔΔCT^ method, and the RT-qPCR results were compared with the transcriptome data. GraphPad Prism 10 was used to draw the graphics.

## 5. Conclusions

This study first reported the characteristics of the *BTB* gene superfamily in *A. sinensis*. A total of 170 *AsBTB* genes were divided into 13 gene families and were unevenly distributed across 16 chromosomes. Tandem duplications and segmental duplications were equally important for *AsBTB* gene amplification. Analysis of the cis-acting elements in the promoter region of *AsBTB* genes revealed its extensive involvement in the cell cycle, embryonic development, cell differentiation, transcriptional regulation, oxidative stress, and ubiquitination regulation. The protein interactions of the *AsBTB* genes primarily centered around CLU-3, mainly functioning through the ubiquitination pathway. Transcriptome and RT-qPCR analyses revealed that *AsBTB* genes are differentially expressed during early sex differentiation and gonadal development in *A. sinensis*, exhibiting multiple expression patterns. The results of this study provide valuable insights into the evolution of the *BTB* gene superfamily. This study also lays a foundation for future research on the roles of *BTB* genes in sex differentiation and early gonadal development in *A. sinensis*.

## Data Availability

Data are contained within the article or Supplementary Material. The genome assembly and annotation files are available at the Genome Warehouse (GWH) under the BioProject PRJCA008589 and c (https://doi.org/10.6084/m9.figshare.24270205.v1) [111]. The transcriptome data remain unpublished [125].

## Supplementary Materials

The following supporting information can be downloaded at https://www.mdpi.com/article/10.3390/ijms251910771/s1.

## Abbreviations

AA	protein length
ANK	ankyrin repeat
*As*	*Alligator sinensis*
ATS1	alpha-tubulin suppressor ATS1 and related RCC1 domain-containing
BACK	BTB and C-terminal Kelch
BBK	BTB-BACK-Kelch
BBP	BTB-BACK-PHR
BTB/POZ	bric-a-brac/tramtrack/broad complex/poxvirus and zinc finger
bZIP	basic leucine zipper
CDS	coding sequence
CUL-3	cullin-3
CySCs	somatic cyst stem cells
DEGs	differentially expressed genes
FPKM	fragments per kilobase million
GCL	germ cell-less protein
GSCs	germline stem cells
KCTD	potassium channel tetramerization domain-containing
KELCH	Kelch repeat
LSE	lineage-specific expansion
MATH	meprin and TRAF-C homology
MW	molecular weight
PGC	primitive germ cell
PHR	photolyase-homologous region
Pi	isoelectric point
POK	POZ and Krüppel-like zinc finger
RT-qPCR	quantitative real-time polymerase chain reaction
T1-Kv	voltage-gated potassium channel T1
UTR	untranslated region
ZF	zinc finger
