# Supplementary material for: Genome-Wide Identification and Expression Analysis of the BTB Gene Superfamily Provides Insight into Sex Determination and Early Gonadal Development of Alligator sinensis
Source: Int J Mol Sci. 2024 Oct 7;25(19):10771. doi: 10.3390/ijms251910771 (PMC11477308; doi:10.3390/ijms251910771)
Supplement: Supplementary file 1 [file ijms-25-10771-s001.zip › Additional file 2.pdf]

Table S2 The *BTB* superfamily genes in *Alligator sinensis*

Name	Identifier	Chr	Genomic position	CDS	AA	MW(Da)	pI	Classification	Subcellular localization
AsBTB1	EVM0003215.1	1	18929449-18963960	10	392	44458.00	6.99	MATH-BTB	nucl
AsBTB2	EVM0003720.1	1	40965144-40975532	6	606	68190.11	5.09	BTB-BACK-Kelch	cyto
AsBTB3	EVM0017005.1	1	41097225-41108844	3	714	78307.31	8.64	BTB-BACK-Kelch	mito
AsBTB4	EVM0019218.1	1	64740317-64748352	6	435	48478.28	6.53	BTB-KCTD	nucl
AsBTB5	EVM0018049.1	1	86181357-86205772	10	374	42150.43	5.58	MATH-BTB	nucl
AsBTB6	EVM0002960.1	1	92237106-92251176	5	605	68594.85	5.84	BTB-BACK-Kelch	cyto
AsBTB7	EVM0014150.1	1	92255307-92259377	2	692	78095.65	5.83	BTB-BACK-Kelch	cyto
AsBTB8	EVM0008091.2	1	106127863-106146710	5	586	66260.29	5.24	BTB-BACK-Kelch	nucl
AsBTB9	EVM0001479.1	1	111730632-111732217	2	510	56841.11	5.84	T1-KV	plas
AsBTB10	EVM0001985.1	1	111936229-111944217	2	320	37191.97	4.88	T1-KV	plas
AsBTB11	EVM0012324.1	1	112080272-112083608	1	522	57377.39	4.96	T1-KV	plas
AsBTB12	EVM0005281.1	1	114810713-114833936	3	505	56651.38	5.56	BTB-BACK-Kelch	cyto
AsBTB13	EVM0013698.1	1	149937473-150007641	14	599	67177.24	5.98	ANK-BTB-BACK	cyto
AsBTB14	EVM0014200.3	1	153071655-153081377	5	246	28390.25	5.83	BTBonly	nucl
AsBTB15	EVM0002864.1	1	178979222-178979988	2	189	20505.85	4.62	T1-KV	extr
AsBTB16	EVM0021179.1	1	206458728-206465329	6	537	61072.52	6.72	T1-KV	plas
AsBTB17	EVM0001355.1	1	249986174-249989303	4	560	61922.38	8.94	BTB-BACK-Kelch	plas
AsBTB18	EVM0019376.1	2	15247897-15345002	11	622	73141.23	7.14	BTB-BACK-Kelch	plas
AsBTB19	EVM0016761.1	2	21555840-21557737	2	505	58166.79	5.11	T1-KV	plas
AsBTB20	EVM0020062.1	2	28615247-28618926	1	489	55731.71	6.15	T1-KV	plas
AsBTB21	EVM0002007.1	2	41470940-41473795	2	322	34427.81	5.55	BTBonly	nucl
AsBTB22	EVM0018750.1	2	47583832-47604501	4	634	70828.53	6.44	BTB-BACK-Kelch	nucl
AsBTB23	EVM0013357.1	2	70164371-70258948	29	1339	149015.32	6.74	ANK-BTB-BACK	extr
AsBTB24	EVM0013544.1	2	76519629-76550667	4	754	83493.70	5.11	BTB-bZIP	nucl
AsBTB25	EVM0000781.4	2	93975442-93991592	8	783	88511.88	8.45	BTB-ZF	nucl
AsBTB26	EVM0015308.1	2	129311543-129320692	3	508	56754.90	5.60	BTB-ZF	nucl
AsBTB27	EVM0000707.1	2	159211132-159215503	2	531	59577.74	5.37	BTB-ZF	nucl
AsBTB28	EVM0006113.1	2	170296755-170330450	11	619	70009.15	5.97	BTBonly	nucl
AsBTB29	EVM0005167.1	2	186343669-186397762	18	814	88673.01	6.86	BTB-KCTD	nucl
AsBTB30	EVM0008827.1	2	193011590-193041183	2	419	47375.43	8.29	T1-KV	plas
AsBTB31	EVM0005590.4	2	220681457-220694921	5	529	59076.42	8.05	BTB-BACK-PHR	nucl
AsBTB32	EVM0006838.1	2	225838389-225895253	14	1817	206207.72	8.24	BTB-ZF	nucl
AsBTB33	EVM0010219.1	3	6892131-6939311	5	683	75557.25	6.13	BTB-ZF	nucl
AsBTB34	EVM0012252.1	3	20973409-21009643	11	1047	119093.29	8.08	BTB-ZF	nucl
AsBTB35	EVM0003049.1	3	48211601-48243937	3	694	76520.92	4.62	BTB-bZIP	nucl
AsBTB36	EVM0010345.2	3	57259167-57288581	2	1056	118973.33	8.50	BTB-ZF	nucl
AsBTB37	EVM0014914.1	3	74319806-74344593	3	626	72238.56	6.26	BTB-BACK-Kelch	cyto
AsBTB38	EVM0010801.1	3	76080319-76099506	3	597	67747.70	6.13	BTB-BACK-Kelch	mito
AsBTB39	EVM0007708.1	3	171215928-171216701	1	257	29628.96	6.61	BTBonly	cyto
AsBTB40	EVM0012300.1	3	173797795-173868905	13	552	60403.42	5.87	ATS1-BTB-BACK	nucl

AsBTB41	EVM0006443.1	3	174743834-174773762	11	531	58272.15	5.90	ATS1-BTB-BACK	nucl
AsBTB42	EVM0007877.1	3	175081315-175084398	2	277	31588.68	6.00	BTBonly	cyto
AsBTB43	EVM0007216.1	3	199149569-199159987	2	614	69929.92	5.50	BTB-BACK-Kelch	cyto
AsBTB44	EVM0005678.1	3	217880841-217896843	2	260	29749.01	5.89	BTB-KCTD	cyto-nucl
AsBTB45	EVM0004082.1	3	217968394-217977301	3	504	56373.00	5.30	BTBonly	cyto
AsBTB46	EVM0001097.1	3	224605046-224616247	7	591	65713.20	5.93	BTB-BACK-Kelch	plas
AsBTB47	EVM0019847.1	4	3373974-3422163	10	578	64279.31	5.47	BTB-BACK-Kelch	nucl
AsBTB48	EVM0001961.1	4	6911076-6924593	6	619	70150.45	4.97	BTB-BACK-Kelch	cyto
AsBTB49	EVM0010277.1	4	6935391-6971068	6	776	87936.47	5.59	BTB-ZF	nucl
AsBTB50	EVM0001367.2	4	61657422-61691764	12	586	66073.76	6.08	BTB-BACK-Kelch	nucl
AsBTB51	EVM0005080.1	4	90946000-90950524	3	619	70721.82	5.66	BTB-BACK-Kelch	cyto
AsBTB52	EVM0018490.1	4	144187932-144194363	2	709	76205.21	5.99	BTB-ZF	nucl
AsBTB53	EVM0017737.3	4	154038440-154068890	11	906	104078.57	8.02	BTB-ZF	nucl
AsBTB54	EVM0000809.1	4	164241936-164253903	13	641	71671.18	5.28	BTB-BACK-Kelch	plas
AsBTB55	EVM0004207.1	4	171902382-171909335	4	519	57815.00	6.16	BTB-ZF	nucl
AsBTB56	EVM0004025.1	4	171972325-171997100	11	609	67933.52	6.45	BTB-BACK-Kelch	nucl
AsBTB57	EVM0015156.1	4	179893530-179905292	2	485	55336.02	6.10	T1-KV	plas
AsBTB58	EVM0015078.1	4	217071015-217085971	8	582	64713.04	5.36	BTB-BACK-Kelch	cyto-nucl
AsBTB59	EVM0004634.1	4	217793221-217798439	8	490	51592.18	9.47	BTBonly	extr
AsBTB60	EVM0001396.1	5	3442205-3467962	8	518	58338.69	6.12	BTB-BACK-Kelch	cyto
AsBTB61	EVM0013298.1	5	25161581-25166696	2	762	80327.33	4.93	BTBonly	nucl
AsBTB62	EVM0015808.1	5	60035847-60041890	2	667	74147.30	5.04	T1-KV	plas
AsBTB63	EVM0005111.1	5	70011380-70083140	17	792	88693.59	5.63	ANK-BTB-BACK	mito
AsBTB64	EVM0013168.1	5	81258094-81267033	5	519	58286.14	5.46	BTB-BACK-Kelch	nucl
AsBTB65	EVM0009055.2	5	121040887-121047071	2	473	52619.90	5.94	BTB-ZF	nucl
AsBTB66	EVM0019360.1	5	123012487-123014730	4	534	59539.37	7.87	BTB-BACK-PHR	mito
AsBTB67	EVM0012147.6	5	123219401-123239960	2	710	81987.28	6.15	BTB-ZF	nucl
AsBTB68	EVM0017934.2	5	123247841-123258933	3	437	49145.96	8.19	BTBonly	nucl
AsBTB69	EVM0002291.1	5	132959784-132980044	5	574	64606.78	5.48	BTB-BACK-Kelch	plas
AsBTB70	EVM0008073.1	5	152349793-152404832	10	1121	125952.03	6.52	BTBonly	nucl
AsBTB71	EVM0006845.2	6	634079-639658	8	723	80174.47	8.11	RhoBTB	plas
AsBTB72	EVM0019994.1	6	27985635-28009447	7	769	85803.96	6.37	BTB-ZF	nucl
AsBTB73	EVM0002316.1	6	53711994-53713219	2	380	40997.28	8.45	BTB-KCTD	nucl
AsBTB74	EVM0002160.1	6	86011079-86107926	8	368	41106.68	8.79	BTB-BACK-Kelch	plas
AsBTB75	EVM0014480.1	7	2990942-2992717	1	591	66556.55	8.04	BTB-BACK-Kelch	nucl
AsBTB76	EVM0018161.1	7	4908647-4933636	2	715	78548.59	6.17	BTB-ZF	nucl
AsBTB77	EVM0016852.1	7	60692457-60705934	3	448	50905.14	5.74	BTB-ZF	nucl
AsBTB78	EVM0013522.1	7	67968651-67981067	5	274	31285.66	8.18	BTBonly	cyto
AsBTB79	EVM0014903.3	7	72399074-72409448	2	376	41063.30	8.08	BTBonly	nucl
AsBTB80	EVM0009340.1	7	99246052-99286768	6	615	68322.14	4.89	BTB-ZF	nucl
AsBTB81	EVM0000316.1	7	112687614-112689047	1	477	54160.16	5.43	T1-KV	plas
AsBTB82	EVM0017809.1	7	122052513-122065553	4	506	56863.60	4.93	T1-KV	plas
AsBTB83	EVM0019998.1	7	132668324-132681214	4	580	66716.78	6.44	BTB-BACK-Kelch	plas
AsBTB84	EVM0010172.1	8	12534486-12550356	2	606	69080.70	5.23	BTB-ZF	nucl

AsBTB85	EVM0015293.1	8	28767759-28801386	7	621	71443.69	4.92	BTB-ZF	nucl
AsBTB86	EVM0018545.1	8	49472875-49479165	3	589	66043.74	6.53	BTB-BACK-Kelch	mito
AsBTB87	EVM0009180.1	8	64215437-64263427	13	642	72585.06	7.03	RhoBTB	plas
AsBTB88	EVM0012733.1	8	112021226-112032157	2	570	65664.09	6.39	T1-KV	plas
AsBTB89	EVM0009128.1	9	2943807-2974266	13	1581	175416.86	5.32	BTBonly	nucl
AsBTB90	EVM0005623.1	9	38620680-38648195	7	208	23764.20	6.24	BTBonly	cyto
AsBTB91	EVM0016411.1	9	39729779-39759403	17	1235	136753.29	5.60	BTB-ZF	nucl
AsBTB92	EVM0016080.1	9	40159122-40165082	5	626	71763.84	9.00	BTB-BACK-Kelch	cyto
AsBTB93	EVM0015625.2	9	41090869-41115872	16	808	88898.73	5.98	BTB-ZF	nucl
AsBTB94	EVM0004340.1	9	47494104-47525703	12	590	65032.91	7.07	BTB-BACK-Kelch	nucl
AsBTB95	EVM0016217.1	9	56608768-56620410	10	699	79303.30	8.77	BTB-ZF	nucl
AsBTB96	EVM0007677.1	9	56623685-56634000	4	589	65632.76	5.41	BTB-BACK-Kelch	extr
AsBTB97	EVM0009868.1	9	65179962-65190035	9	548	62218.91	8.54	T1-KV	plas
AsBTB98	EVM0005392.1	9	74029923-74033900	1	574	63962.71	5.41	T1-KV	plas
AsBTB99	EVM0010621.1	9	74096263-74097762	1	499	56636.68	4.79	T1-KV	plas
AsBTB100	EVM0003488.1	9	74206539-74208101	1	520	59194.75	4.95	T1-KV	plas
AsBTB101	EVM0002440.1	9	74542568-74563049	4	594	65816.67	6.55	T1-KV	plas
AsBTB102	EVM0019413.1	9	75970370-76011634	12	564	63257.93	5.17	BTB-BACK-Kelch	cyto-nucl
AsBTB103	EVM0015229.1	9	76205511-76256400	20	790	90062.84	6.21	BTB-BACK-Kelch	cyto
AsBTB104	EVM0020542.2	9	83194704-83236713	25	1215	134219.11	5.71	ANK-BTB-BACK	cyto-nucl
AsBTB105	EVM0005409.2	9	87623062-87635841	4	333	38061.52	5.95	BTBonly	cyto
AsBTB106	EVM0010108.1	9	90194014-90207792	7	313	35850.04	7.95	BTBonly	mito
AsBTB107	EVM0008442.1	9	96883561-96889000	2	656	71186.74	6.53	BTB-ZF	nucl
AsBTB108	EVM0011499.1	9	99628745-99636192	6	628	68315.70	5.48	BTBonly	plas
AsBTB109	EVM0015827.1	9	107131344-107182902	7	616	68476.57	5.64	BTB-ZF	nucl
AsBTB110	EVM0001999.1	9	117187904-117200032	2	511	58638.26	8.01	T1-KV	plas
AsBTB111	EVM0009094.1	9	119573574-119605140	9	377	42040.93	5.53	BTB-BACK-PHR	cyto
AsBTB112	EVM0001274.1	9	121445271-121468613	3	546	60724.83	4.85	BTB-ZF	nucl
AsBTB113	EVM0017895.1	9	125823930-125849457	2	542	61286.77	5.45	BTB-BACK-Kelch	plas
AsBTB114	EVM0000989.1	9	129876617-129877870	1	417	44897.60	5.25	BTB-ZF	nucl
AsBTB115	EVM0002249.1	9	133789073-133791202	1	709	77023.27	6.14	BTB-ZF	nucl
AsBTB116	EVM0010015.1	9	138872520-138891731	5	773	85329.50	8.06	BTB-ZF	nucl
AsBTB117	EVM0016595.1	9	148256330-148258198	1	622	67207.37	5.64	BTB-KCTD	cyto-nucl
AsBTB118	EVM0010420.1	9	148282449-148287394	8	447	47186.09	9.37	BTB-ZF	nucl
AsBTB119	EVM0014984.2	9	148546054-148555416	3	1130	120524.51	8.64	BTB-ZF	nucl
AsBTB120	EVM0018602.1	10	2158146-2212864	8	671	76532.04	7.49	RhoBTB	plas
AsBTB121	EVM0012280.1	10	44903913-44912637	4	478	53762.10	5.15	BTB-ZF	nucl
AsBTB122	EVM0012784.1	10	44934622-44942810	5	509	57730.51	5.98	BTB-ZF	nucl
AsBTB123	EVM0018065.1	10	44952577-44963561	3	417	47151.33	8.84	BTBonly	nucl
AsBTB124	EVM0005588.1	10	45834608-45843608	3	679	74985.47	6.45	BTB-BACK-Kelch	nucl
AsBTB125	EVM0002844.1	10	66703243-66754120	17	614	70164.17	9.12	BTBonly	cyto
AsBTB126	EVM0015712.1	11	1417782-1439214	8	588	66679.18	5.66	BTB-BACK-Kelch	nucl
AsBTB127	EVM0004949.1	11	5241120-5273910	7	610	69415.35	5.38	BTB-BACK-Kelch	cyto
AsBTB128	EVM0014886.1	11	5312358-5323246	6	600	68141.90	6.02	BTB-BACK-Kelch	plas

AsBTB129	EVM0017553.1	11	19299931-19332495	5	1196	135129.96	8.34	BTB-ZF	nucl
AsBTB130	EVM0007806.1	11	37982666-37998663	8	614	69208.06	8.57	BTB-ZF	nucl
AsBTB131	EVM0017388.1	11	49840454-49860461	10	707	79005.23	8.22	BTB-ZF	nucl
AsBTB132	EVM0016651.1	11	61677228-61680461	4	793	88202.63	5.71	BTB-BACK-Kelch	extr
AsBTB133	EVM0001646.3	11	74693162-74723019	14	513	58088.24	7.87	BTBonly	nucl
AsBTB134	EVM0002387.1	11	79991317-80013605	11	759	86234.58	6.69	RhoBTB	nucl
AsBTB135	EVM0011703.1	11	81001580-81013949	11	357	38991.47	6.26	BTBonly	mito
AsBTB136	EVM0004473.2	12	12089212-12100851	2	340	38217.41	8.47	BTB-KCTD	plas
AsBTB137	EVM0011368.1	12	18549185-18559085	8	638	69491.28	7.89	T1-KV	plas
AsBTB138	EVM0014955.3	12	24083457-24094133	5	521	56284.90	5.30	BTB-BEN	nucl
AsBTB139	EVM0010574.1	12	24562685-24566050	5	589	65296.94	5.59	BTB-BACK-Kelch	cyto
AsBTB140	EVM0014012.1	12	34695468-34704802	6	158	18108.34	4.75	BTBonly	cysk
AsBTB141	EVM0009787.1	12	48550778-48639788	8	787	88360.30	6.75	BTB-BACK-Kelch	extr
AsBTB142	EVM0013685.1	12	57798005-57800295	1	677	74621.32	4.86	BTB-ZF	nucl
AsBTB143	EVM0009686.1	12	67238544-67241609	2	430	50062.98	7.15	T1-KV	plas
AsBTB144	EVM0011402.1	12	75933538-75958907	10	614	68531.65	6.00	BTB-BACK-Kelch	nucl
AsBTB145	EVM0016132.1	13	5447649-5458227	5	676	76481.56	6.25	BTB-BACK-Kelch	cyto
AsBTB146	EVM0004662.1	13	5732206-5752286	2	510	58266.13	5.43	T1-KV	plas
AsBTB147	EVM0005545.1	13	7713624-7757390	11	597	67742.51	5.80	BTB-BACK-Kelch	plas
AsBTB148	EVM0005842.1	13	45277511-45342748	17	935	108162.30	5.69	T1-KV	cyto
AsBTB149	EVM0016951.1	13	57413138-57442671	7	616	67244.52	8.74	BTB-ZF	nucl
AsBTB150	EVM0013900.1	13	58952497-58976451	7	638	73031.66	5.21	BTB-BACK-Kelch	plas
AsBTB151	EVM0012958.1	13	60974585-60990084	2	570	62573.53	6.51	BTB-ZF	nucl
AsBTB152	EVM0010242.2	14	7060168-7126663	15	586	64969.11	5.37	BTB-BACK-Kelch	nucl
AsBTB153	EVM0013610.1	14	14411892-14416527	2	337	38916.10	9.03	BTB-KCTD	cyto
AsBTB154	EVM0004891.1	14	55324310-55345951	3	484	55157.96	5.23	T1-KV	plas
AsBTB155	EVM0001360.1	14	59676315-59689732	3	597	68190.04	8.03	T1-KV	plas
AsBTB156	EVM0001388.1	14	69761322-69814038	2	455	50046.08	4.96	BTB-FYVE	nucl
AsBTB157	EVM0009874.1	15	742032-744167	2	237	27704.44	5.83	BTBonly	cyto
AsBTB158	EVM0019474.1	15	4647872-4655791	4	608	69568.66	6.62	BTB-BACK-Kelch	cyto
AsBTB159	EVM0002813.1	15	18588561-18627391	12	477	54615.54	4.81	ANK-BTB-BACK	cyto
AsBTB160	EVM0005563.1	15	18763984-18796516	5	635	71736.29	7.45	BTB-BACK-Kelch	cyto
AsBTB161	EVM0015085.1	15	32965534-32983005	2	499	55494.88	6.03	BTB-ZF	nucl
AsBTB162	EVM0000188.1	15	32992160-32998404	2	521	58549.13	5.80	BTB-ZF	nucl
AsBTB163	EVM0014976.1	15	35340626-35341948	1	440	49811.26	6.12	BTB-ZF	nucl
AsBTB164	EVM0001755.1	15	35347199-35352483	2	427	48775.39	6.44	BTB-ZF	nucl
AsBTB165	EVM0020568.1	15	36731742-36752085	5	578	63182.27	5.64	BTB-BEN	nucl
AsBTB166	EVM0006684.1	15	49256812-49265930	4	620	69565.81	8.55	BTB-BACK-Kelch	pero
AsBTB167	EVM0012242.1	16	7237221-7239133	1	521	55737.51	5.90	BTB-ZF	nucl
AsBTB168	EVM0008857.1	16	13750715-13754743	2	569	61088.27	6.71	BTB-ZF	nucl
AsBTB169	EVM0014544.3	16	49109520-49121873	3	611	68631.51	6.04	BTB-BACK-Kelch	cyto-nucl
AsBTB170	EVM0008033.1	16	55486173-55490281	1	479	53767.30	5.30	BTB-BACK-Kelch	plas

Note: Nucl: nucleus, cyto: cytoplasm, mito: mitochondria, plas: plasma membrane, extr: extracellular, cyto-nucl: cytoplasm-nucleus, 154 cysk: cytoskeleton, pero: peroxisome.
