# Supplementary material for: Genome-Wide Identification and Expression Analysis of the BTB Gene Superfamily Provides Insight into Sex Determination and Early Gonadal Development of Alligator sinensis
Source: Int J Mol Sci. 2024 Oct 7;25(19):10771. doi: 10.3390/ijms251910771 (PMC11477308; doi:10.3390/ijms251910771)
Supplement: Supplementary file 1 [file ijms-25-10771-s001.zip › Additional file 6.pdf]

Table S6 Sequences of 30 predicted motifs of AsBTB proteins

Motif	Width	Motif Sequence
1	15	TGEKPYKCEICGKSF
2	21	EFPAHKAYLAACSPYFRAMFT
3	15	HGVAVLGGKLYVYGG
4	15	VECYDPETNEWTEVA
5	29	PSHSSSLLQQLNEQRLQGLLCDVTLYYEG
6	27	EAFEALLDFLYTGKLRLSEENVEEVLE
7	57	IPDAFWWAVVTMTTVGYGDMVPYTYGGKIVGSLCAIAGVLTIALPVPVIVSNFSYFY
8	50	DPFFIVETVCIIWFTFELLVRFIACPSKCKFFKNIMNIIDIVAILPYYIT
9	100	RVVINISGLRFETQLKTLAQFPETLLGDPRKRMRYFDPLRNEYFFDRNRPSFDAILYYYQSGGRLRRPVNVPID IFSEEIRFYQLGEEAMEKFREDEGFI
10	21	AASYLQMPDVVEACCEFLKSQ
11	41	RLVRVFRIFKLSRHSKGLRILGYTLKASMRELGLLLFFLFV
12	41	RRQVWLLFEYPESSLPARYIAIVSYLFILISIYIFCLETLP
13	28	SDELNYPSEEQVFEAVLRWVKHDRQRRR
14	11	KSNLKRHMRIH
15	18	IITLNVGGIRYTTSRSTL
16	15	DPSNCLGIRAFADTH
17	29	RYILQHFEEYSKTEEFLQLSKDQLIDLLS
18	21	ELLKHVRLPLLPPQYLVETVE
19	36	GGSPHSMALTENGEVYAWGYNGYGQLGTGNTNNQPT
20	100	FSIANPNSLRHVKTMWYPEIKHFCPRTPVILVGCQLDLRYADLEAVNRARRPLARPIKPSDILPPERGREVAKE LGIPYYETSVVAQFGIKDVFDNAIRA
21	21	IRQDPECRDLLIEAMNYHLLP
22	27	EELEYWGIDEEFLDSCCSRRYRERKEE
23	40	AMFSGRRPLARDSEGRFFIDRDGFLFRYILDYLRDSQLVL
24	65	MDTDMDYERPNVETIKCVVVGDNAVGKTRLICARACNATLTQYQLLATHVPTVWAIDQYRVCQEV
25	21	GVILFSSAVYFAEKDEPSTLF
26	15	DYDVETNEYFFDRDP
27	29	DKRGQTPLHLAIQHGNEEIVSLLLEAGAD
28	21	VWCYSSETNQWILVPPMNKAR
29	81	ANRLCLPHLVALTEQYAVTELTRASVQQVEIDGDVLVYLEMAQFHNAYQLAAECLHYICTNYNSVCRKFPRE MLAMSAGNQ
30	57	AELLEVFDLRMMVANILNKEAFMNQEITKAFHVRRANRYKECLAKGTFSDVTFKLDD
